# How to improve public health literacy based on polycentric public goods theory: preferences of the Chinese general population

**DOI:** 10.1186/s12889-022-13272-z

**Published:** 2022-05-09

**Authors:** Yaxin Gao, Li Zhu, Zi Jun Mao

**Affiliations:** 1 Department of Gastrointestinal Surgery, Tongji Hospital, Tongji Medical College, Huazhong University of Science and Technology, Jie Fang Ave, Wuhan, No. 1095 China; 2 College of Public Administration, Huazhong University of Science and Technology, No 1037 Luau Road, Hongshan District, Wuhan, 430074 China; 3 Non-traditional Security Institute, Huazhong University of Science and Technology, Wuhan, 430074 China

**Keywords:** Health literacy, Public goods, Multi-center supply, Health communication, Social media, Social equity, Critical thinking

## Abstract

**Background:**

In the current era of big data, it is critical to address people’s demand for health literacy. At present, the traditional mode of communicating scientific health knowledge and information technology is interchangeable, resulting in the emergence of a new mode of communicating health literacy. To publicize health education and health literacy in a targeted way, to meet the public’s needs, and to understand how the public’s demand for subjects, contents, and forms of health literacy service has changed in the era of COVID-19, the investigation of public’s demand for health information and health literacy was conducted.

**Objective:**

This study aims to understand the differences in demand for health literacy service providers, contents, channels, forms, and facilities among Chinese citizens with different genders, ages, education levels, economic conditions, and living environments, and to provide reasonable recommendations for developing public health literacy.

**Methods:**

Questionnaire Star was used to conduct a large sample of random online surveys. In Wuhan, Hubei Province, 2184 questionnaires were issued, 8 invalid questionnaires were eliminated, and 2176 were recovered, with an effective rate of 99.6%. IBM SPSS Statistics 20 was utilized to analyze the survey data.

**Results:**

(1) In health literacy service providers selected by the public, the proportion of government departments or government collaboration with other institutions exceeded 73%, indicating that health literacy services are public goods; (2) access to health literacy services was lower in township areas than in urban areas (*P* < 0.001, 3) internet media and communicating with acquaintances, which have the highest popularity rate, were also the two channels that were least trusted by the public; and (4) the differences in contents and service channels of health literacy among residents with different genders, ages, education levels, economic status, and living environments were statistically significant.

**Conclusions:**

(1) It is recommended to establish an integrated health literacy service model with multi-center supply. Government departments, medical institutions, and media should cooperate effectively to provide health literacy services. (2) The government should pay attention to the fairness of health education and strengthen the supply of health literacy services in township areas. (3) It is critical to strengthen the public’s ability to discriminate network information and pay attention to scientific thinking cultivation. (4) Health literacy service providers must focus on the differences between public demands and improve the connotation of health literacy services.

**Supplementary Information:**

The online version contains supplementary material available at 10.1186/s12889-022-13272-z.

## Introduction

### Public health literacy

As social economy develops, most groups desire a better life and have a strong demand for health care, but their health literacy has not kept pace [1, 2]. At present, Chinese residents’ health literacy continues to suffer from uneven development and a poor overall level. Urban and rural residents lack the knowledge and skills of maintaining health, such as disease prevention, early detection, emergency rescue, timely medical treatment, rational drug use, emergency risk prevention, etc., and unhealthy lifestyles are prevalent [2]. Public health literacy service aims to enhance public health knowledge, improve public health awareness, effectively prevent various diseases, rationally address health problems, improve the health status of residents, and save social resources and medical costs, it is in need of planned, purposeful and scientific indoctrination of the public, and its effectiveness should be continuously investigated, summarized and analyzed [3]. Singh T proposed that achieving “health for all” requires people to receive health education on immunization, nutrition, first aid and sanitation, with the goal of changing behavior by changing public attitudes and encouraging people to make their own health-related decisions [4].

McCarthy K et al. argued that health literacy promotion requires well-designed, cost-effective and impartial resources that provide consistent information to the public. These resources must overcome the inequality in information access and meet the needs of people with low literacy rate [5]. Jisan Lee et al. put forward that although smart phones and mobile devices have become popular, the accessibility and usability of technology depend on the characteristics of users, these differences may threaten the equity of health information, resulting in medical knowledge asymmetry and unequal access to health literacy service [6].

Song P et al. believed that during the outbreak, based on the effective connection of the Internet and electronic media, most people could easily obtain epidemic-related health information, which was conducive to the control of the epidemic [7]. However, Information overload during the COVID-19 pandemic brought a series of challenges that had never been encountered before, and an “information epidemic” emerged, in which negative news such as false reports, colorism and unscientific knowledge were quickly shared, causing anxiety and pressure among the public and even leading to the loss of life [8]. Some scientists argue that the outbreak creates a tsunami of information, including misinformation and rumors about health, which is quickly amplified by social media [9]. The public health literacy service is now facing the dilemma of “information epidemic” spread with untimely disinformation and dissemination of health science information, weak value leading force of authoritative public institutions, and low relevance of public health literacy promotion methods and contents [10].

### Public goods theory

In 1954, Paul A. Samuelson defined “collective consumer goods” as having noncompetitive properties, meaning that one user’s consumption of the good does not affect another’s consumption of it. In his opinion, only when simultaneous consumption is allowed can it be called public goods [11]. In 1959, Richard Abel Musgrave defined non-competitive and non-exclusive as the attributes that should be possessed to define public goods [12]. In 1991, Robert Wuthnow proposed to establish a coordination mechanism for effective communication between the government, enterprises and the third sector, so that each public goods supplier could give full play to its own advantages and roles to achieve a “win-win” for all parties [13]. In 2000, Janet V. Denhardt, a representative of the new public service theory, criticized the improper involvement of the government as a “coordinator” in the supply of public goods, emphasizing that the role of the government is to serve, rather than manipulate, and proposed the following: the government, citizens, society and market participants should establish a multi-dimensional interaction model of public goods supply, emphasizing attention to and response to citizens’ right to pursue public interests, and maximizing the realization of public interests [14].

Some experts said that after the impact of COVID-19 in 2020, health literacy service embodies obvious public goods attribute. When a major disaster or epidemic occurs, it can quickly stabilize people’s minds and improve the scientific response capacity of the whole society. In daily life, it enables people to have more knowledge and skills in health management [15, 16]. However, there are limited studies on how to improve public health literacy service based on polycentric public goods theory. Due to the difficulties for the government or a single subject to effectively provide public health literacy service and improve public health, especially in the context of the epidemic. This paper aims to address the shortcomings of health literacy services in today’s society. From the perspective of public goods theory, we should think about how to integrate multiple actors, establish a health literacy platform with both scientific and social influence, produce high-quality health literacy products, promote health literacy service to play a real role in society, assist the public in acquiring appropriate health knowledge and skills, and change unhealthy behaviors. Therefore, we should improve the way of providing health literacy service oriented by the demands of residents and improve the integrated service of provision system.

## Methods

To design the questionnaire (Shown in Table 1), we were referred to China Science Popularization Internet Data Report 2020, [17] National Health Insight Report 2021, [18] and Modern Social Survey Method (4th Edition ) [19] and followed the principles of purpose, science, hierarchy, system, operability, and comparability. Questionnaire Star was utilized to conduct a large sample of random online surveys, and the restricted area of filling was Wuhan, Hubei province. A total of 2184 questionnaires were distributed, 8 deemed invalid and were eliminated, and 2176 were recovered, with an effective rate of 99.6%. IBM SPSS Statistics 20 was used to analyze the survey data. Correlation analysis was employed to determine the correlation between each variable (genders, ages, education levels, economic conditions, and living environments) and health literacy channels and contents. The independent sample T-test and multiple regression analysis were used to analyze the differences in health literacy accessibility between urban and rural areas, as well as the factors causing differences. The independent sample T-test and one-way analysis of variance were performed to determine the differences between variables regarding the desire for health literacy.

**Table 1:**
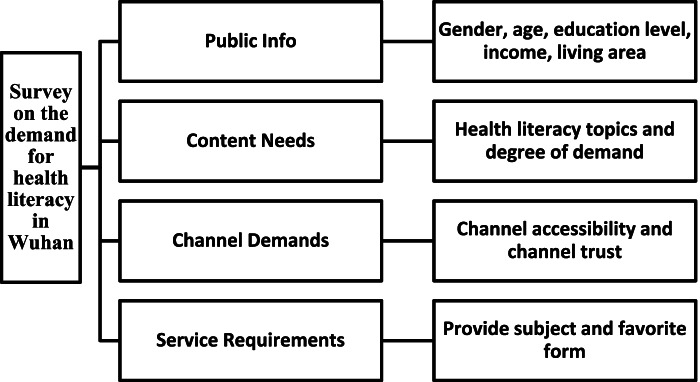
Flow diagram of the questionnaire design

Table 2 describes the baseline characteristics of the survey respondents. The overall effective response rate was 99.6% (2176/2184). 94.5% were between 18 to 60 years old. Eighty-five percent of the respondents were from city, 45% were above bachelor degree and 61.8% of respondents were women.

**Table 2:**
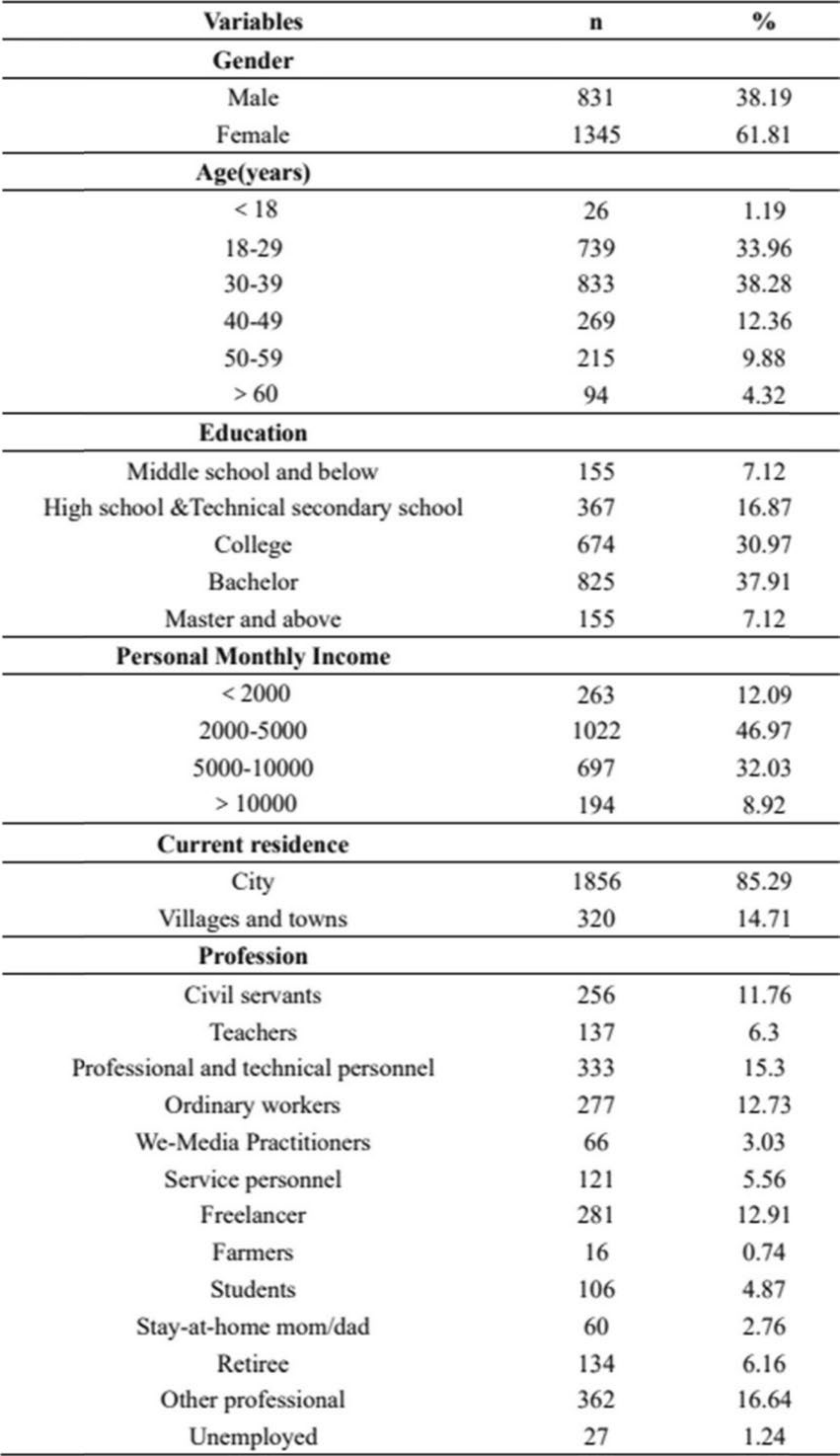
Basic information of respondents

## Results:analysis of health literacy service providers

Due to unclear classification of the scope of health literacy services, unclear understanding of the nature of services, and relevant responsibility subjects, production and provision subjects of services are often confused, resulting in low efficiency of policy implementation and unsatisfactory implementation effect. It meets China’s market economy development criteria by clarifying the primary producers and suppliers of health literacy services. The providers of health services vary according to their nature, especially in services with a high degree of public nature, in which the government holds major responsibility [20]. Producing health literacy services can develop along a broader road, strengthen third sector participation and introduce the private sector, consistent with the direction of market-oriented reform and conducive to healthy development of China’s public health literacy.

The results indicated that 825 people (37.91%) chose government departments, medical institutions, and the media to jointly provide health science services; 771 people (35.43%) residents chose government departments such as communities, and sub-district offices as the main providers of health literacy services, while 454 people (20.86%) chose medical institutions and 126 people (5.79%) chose the media as the main providers of health science services. Additionally, the proportion of Wuhan residents participating in health literacy activities at community health service stations and community public places was the highest, at 50.69 and 46.51%, respectively, (Shown in Table 3).

**Table 3:**
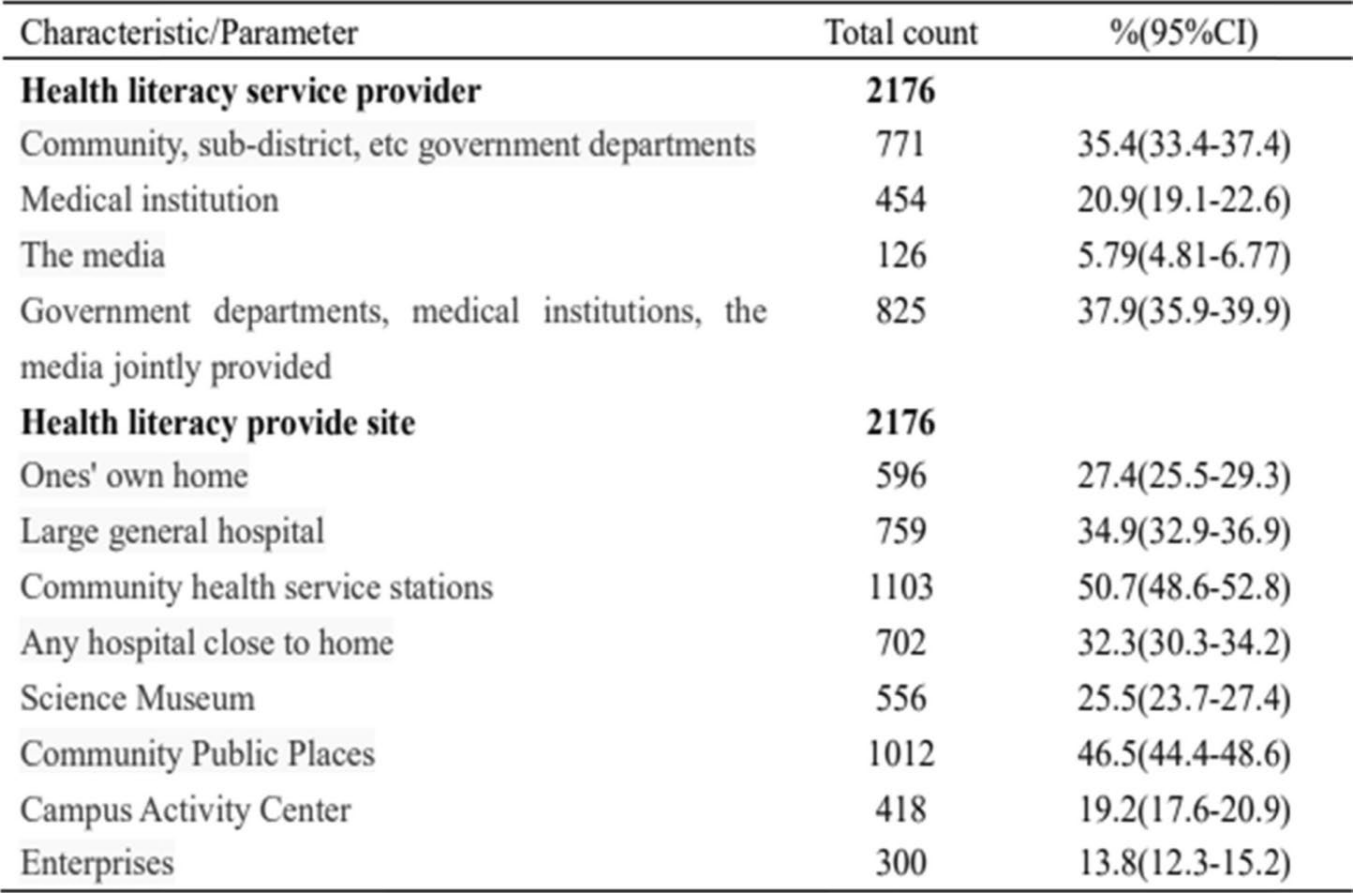
The data analysis of public choice of different health literacy service providers and provide sites

### Analysis of the supply channel and form of health literacy service

Health science is an interdisciplinary field that encompasses medicine, communication, education, and other disciplines. Health literacy channels and forms are inevitably affected by constant changes in information dissemination, and traditional ways of science popularization can no longer adapt to current information environment. Nowadays, Internet has become an essential medium for information transmission and communication, and data processing technology of electronic computers has been widely used in various fields. By following the scientific and technological train, the dissemination mode of health literacy is no longer limited to traditional paper books and TV broadcasts, and the presentation mode of content is no longer limited to text expression [21]. In this environment, the contemporary health literacy communication mode is confronted with revolutionary challenges. Exploring current mainstream health literacy channels and popular forms of health science enables us further improve health literacy service mode, which is oriented to the needs of residents and centered on a new media matrix.

According to survey results, the most important channel for Wuhan citizens to obtain health literacy is Internet media (85.29%), followed by communication with acquaintances (46.88%). WeChat was chosen as the most common online media channel to obtain health science information, with 84.27%. Short videos and articles with pictures were selected as the most popular forms of health literacy by 82.98 and 80.67% of Wuhan citizens, respectively, (Shown in Table 4).

**Table 4:**
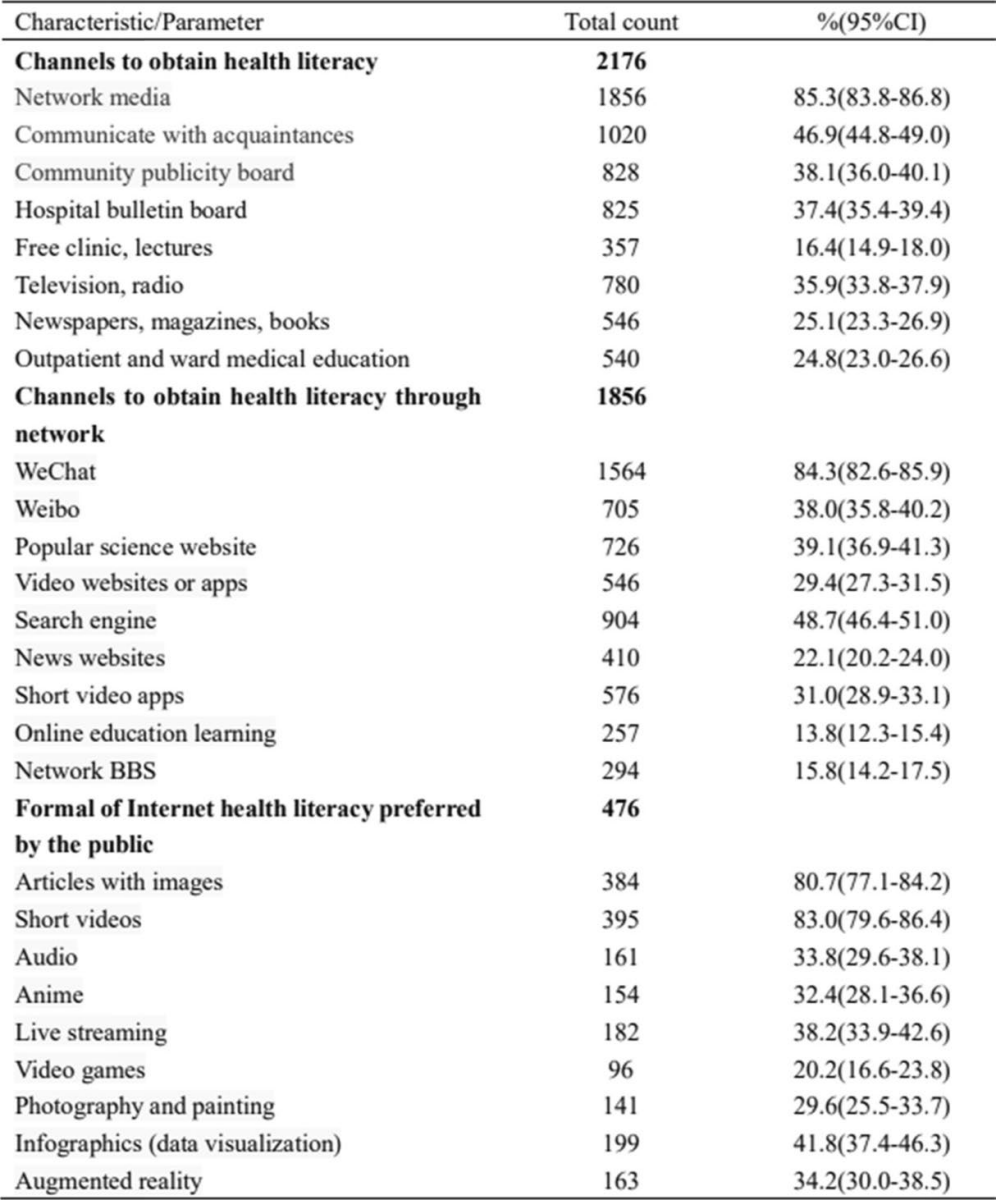
The data analysis of main channels for citizens to obtain health information and formal of Internet health literacy preferred by the public

**Fig. 1:**
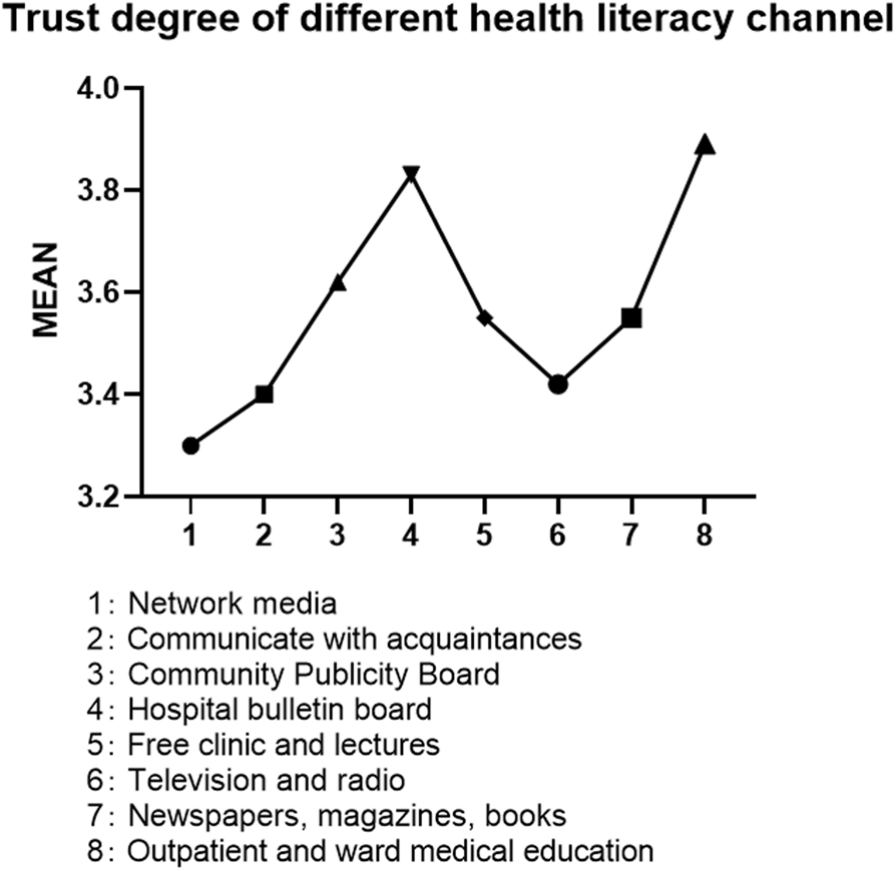
Analysis of trust degree of health literacy channel

The trust degree of health literacy channels was assigned to 5 points for very trust, 4 points for relatively trust, 3 points for general trust, 2 points for relatively distrust, and 1 point for very distrust. Additionally, network media and communication with acquaintances were selected as the two most distrusted health science channels, with scores of 3.3 and 3.4, respectively. The highest score was 3.89 points for outpatient service and medical education inwards and 3.83 points for publicity boards/brochures and electronic screens in medical institutions, (Shown in Fig. 1).

Correlation analyses were conducted between health science channels and demographic characteristics such as gender, age, education level, occupation, and residence. The increase of age was negatively correlated with access to health literacy channels, network media, community, and medical institution bulletin boards but positively correlated with acquaintances, television, and radio channels. Increased education level was positively associated with access to health literacy channels, online media, and hospital publicity boards but negatively correlated with television and radio. Current residence (village) was negatively correlated with access to health litteracy and network media channels, (Shown in Table 5).

**Table 5:**
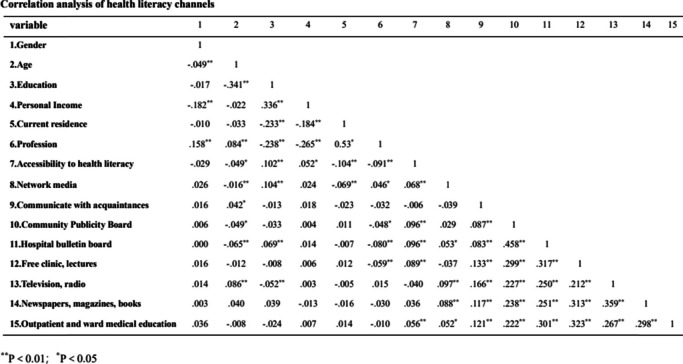
Correlation analysis of health literacy channels

**Fig. 2:**
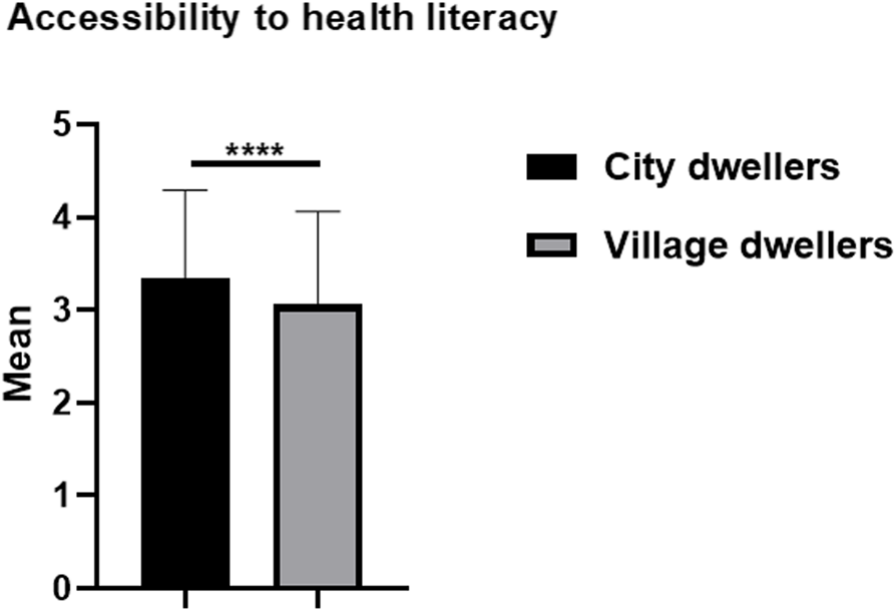
Independent sample T-test on the accessibility to health literacy, taking urban and rural residents as dependent variables. ****p<0.0001

The independent sample T-test was used to analyze the accessibility of health literacy channels. “Always” scored 5 points, “often” scored 4 points, “generally” scored 3 points, “occasionally” scored 2 points, and “never” scored 1 point. The average score of urban residents was 3.34 points, and that of township residents was 3.06 points, *P* < 0.0001, (Shown in Fig. 2).

Utilizing the accessibility of health literacy as the dependent variable and the statistically significant variables in the correlation analysis as the independent variables, the multiple linear regression analysis showed that the current residence (City =1, Town =2), occupation (1 = Civil servants, 2 = Teachers, 3 = Professional and technical personnel, 4 = Ordinary workers, 5 = We-Media Practitioners, 6 = Service personnel, 7 = Freelancer, 8 = Farmers, 9 = Students, 10 = Stay-at-home mom/dad, 11 = Retiree, 12 = Other professional, 13 = Unemployed) and education level (1 = Middle school and below, 2 = High school &Technical secondary school, 3 = College, 4 = Bachelor, 5 = Master and above) were independent factors affecting the accessibility of health science knowledge, (Shown in Table 6).

**Table 6:**
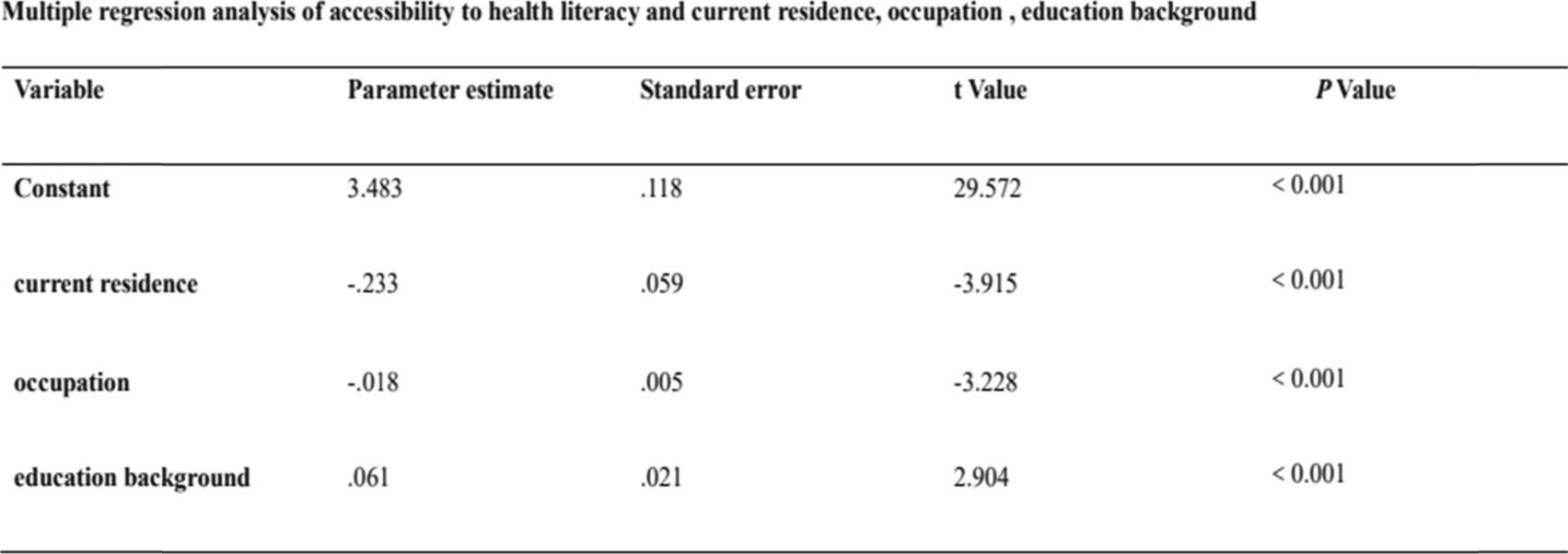
Multiple regression analysis of accessibility of health literacy

### Demand analysis of health literacy content

Health literacy content topics fall into the following categories according to surveillance and prevention, (Shown in Table 7).

**Table 7:** The basis on what health content topic was selected

Topics	Contents	References
Surveillance	First aid Cosmetic surgery Nutrition Mental health Children’ health Facial features Reproductive health Medical technology Traditional Chinese medicine (TCM)	Zideman DA, Singletary EM, Borra V, et al. European Resuscitation Council Guidelines 2021: First aid. Resuscitation. 2021;161:270–290 [22]. Georgiou A, Singh P, Mosahebi A. Plastic surgery and social media in the public health sector. *J Plast Reconstr Aesthet Surg*. 2021;74 (5):1101–1160 [23]. NUTRITION and public health. Nutr Rev. 1950;8 (8):236–237 [24]. Ren FF, Guo RJ. Public Mental Health in Post-COVID-19 Era. Psychiatr Danub. 2020;32 (2):251–255 [25]. Qiao J, Wang Y, Li X, et al. A Lancet Commission on 70 years of women’s reproductive, maternal, newborn, child, and adolescent health in China [26]. Lee DYW, Li QY, Liu J, Efferth T. Traditional Chinese herbal medicine at the forefront battle against COVID-19: Clinical experience and scientific basis. *Phytomedicine*. 2021;80:153337 [27].
Prevention	Tumor Metabolic disease Cardiovascular disease Infectious disease Venereal disease	Liu S, Chen Z, Han L, et al. Integrated multisectoral non-communicable disease prevention and control in China: A review of agencies and policies. J Glob Health. 2020;10 (2):020304 [28]. Xiong WY, Feng ZJ. [Overview on communicable disease surveillance in China]. Zhonghua Liu Xing Bing Xue Za Zhi. 2011 Oct;32 (10):957–60. Chinese [29].

**Fig. 3:**
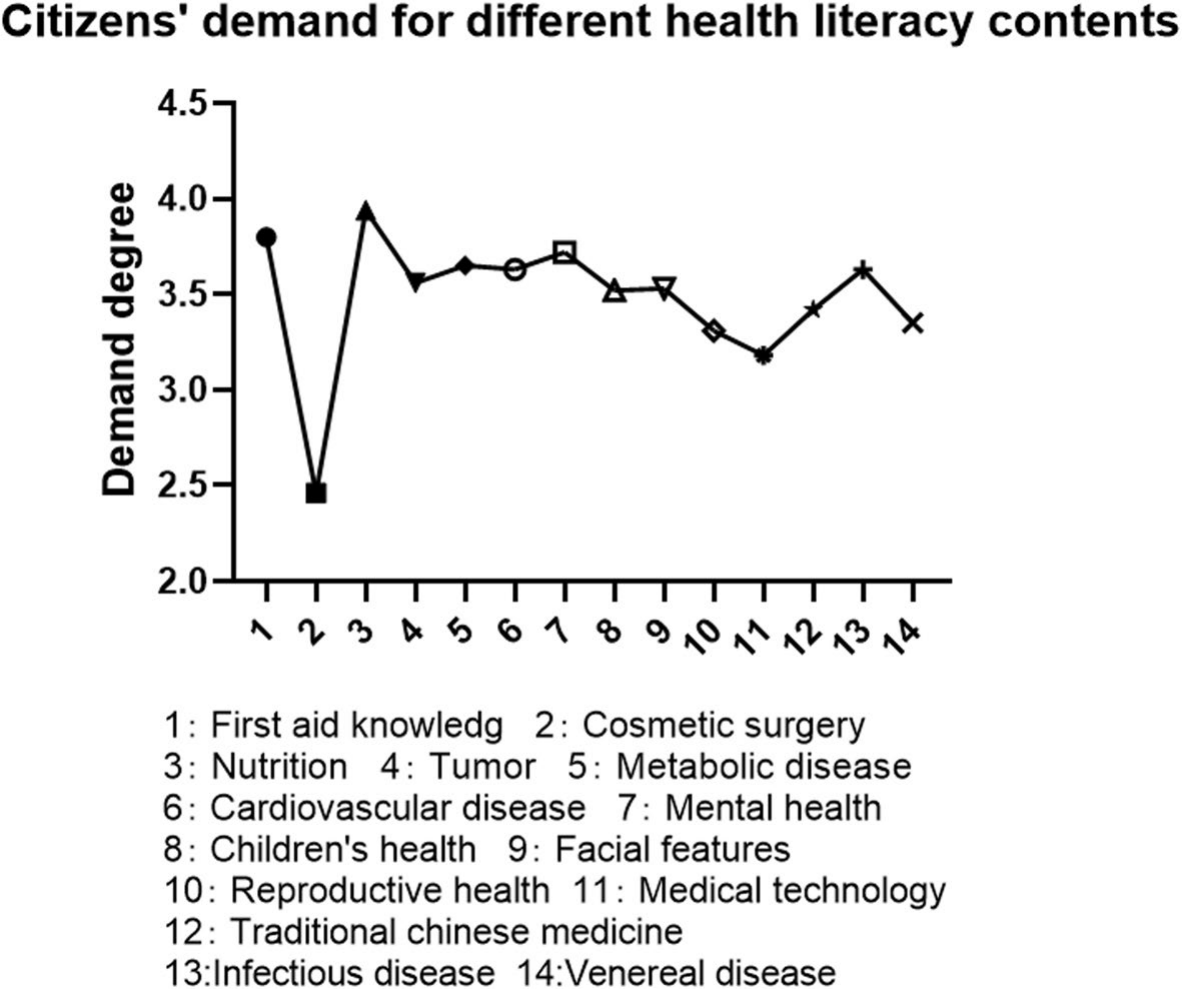
Analysis of citizens’ demand for different health literacy contents

The three most popular health literacy topics with the highest public demand were nutrition, first aid knowledge, and mental health. The value of public health literacy content was assigned according to public’s needs: 5 points for very important, 4 points for comparatively important, 3 points for average, 2 points for little important, and 1 point for no important. The average score of public demand for different health topics in Wuhan was 3.48. The three health topics that most require health literacy are as follows: (1) nutrition (food safety, healthy diet, balanced nutrition, etc.), 3.94 points; (2) first aid knowledge (cardiopulmonary resuscitation, trauma treatment, etc.), 3.8 points; (3) and mental health (emotional expression and control, integrity and harmony of personality, good interpersonal relationship, and sleep), 3.72 points; Public demand for medical technology (3.18 points) and plastic surgery (2.46 points) was low, (Shown in Fig. 3).

Influenced by gender, education, age, occupation, residence, and other factors, the public’s demand for health science content is different, (Shown in Table 8).

**Table 8:**
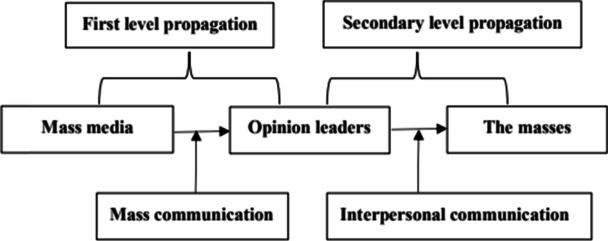
Correlation analysis of health literacy contents

**Fig. 4:**
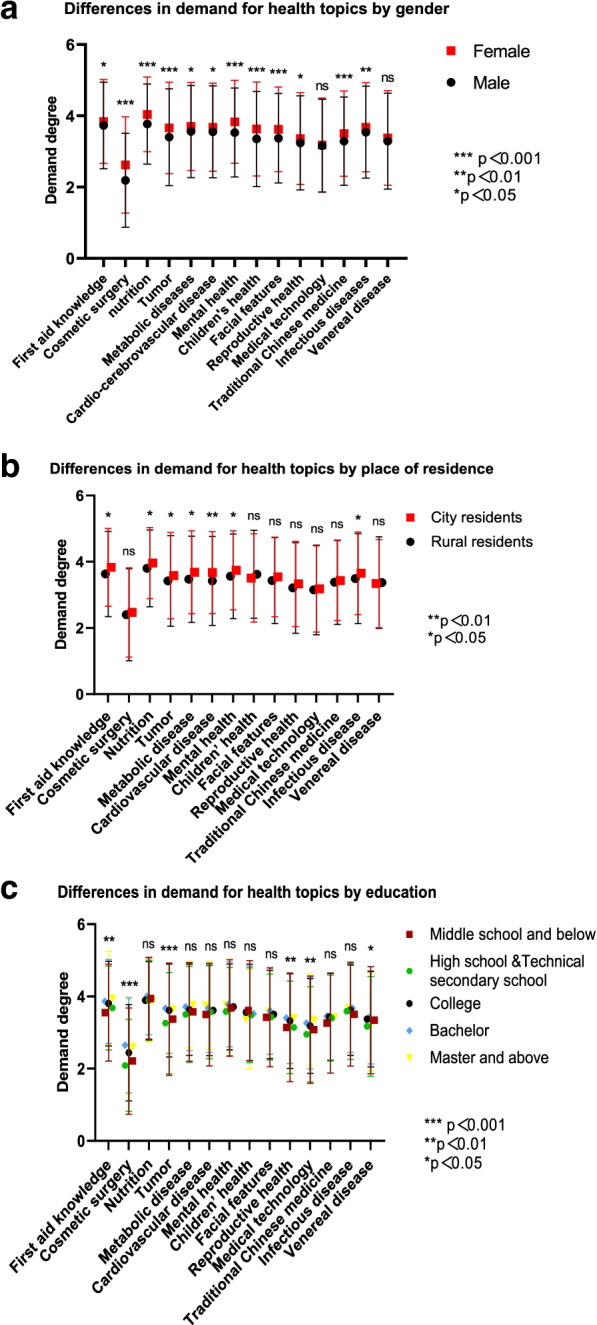
Analysis of the differences in demand for health literacy by different masses. *p<0.05, **p<0.01, *** *p<*0.001, ns: no significance. **a** Analysis of different genders’ demands on health literacy topics. **b**. Analysis of urban and rural residents’ demands for health literacy topics. **c**. Analysis of demands of residents with different education levels on health literacy topics

The demand for health literacy is generally higher among women than men, with statistical differences in all items except medical technology and sexually transmitted diseases (Fig. 4a). In terms of required content on first aid, nutrition, tumor, metabolic diseases, cardiovascular and cerebrovascular diseases, mental health, and infectious diseases, urban residents were higher than rural residents, and the difference was statistically significant (Fig. 4b). As educational background improves, residents’ demand for health literacy of first aid, plastic surgery, tumor, reproductive health, and medical technology increased, and the difference was statistically significant (Fig. 4c).

## Discussion

### Health literacy service has the attribute of public goods

According to survey results, although Wuhan residents have higher trust in the health literacy services provided by professional doctors or medical institutions, the proportion of community, sub-district offices, and other government departments as the main providers of health literacy services (35.43%) is much higher than that of medical institutions (20.86%) or media (5.79%). Health literacy aims to assist individuals and groups in preventing diseases, promoting health, improving the quality of life, and pursuing social public value. It is characterized by universality, public welfare, and fairness, and every citizen has the right to obtain health literacy services.

According to public economics theory, public goods are the products or services that can be consumed or enjoyed by most people, are non-competitive in their consumption or use, and are not excludable in terms of benefits [30]. They are distinguished from private goods enjoyed by individual consumers and are hostile, exclusive, or divisible. Health literacy services have the characteristics of public goods enjoyed by all, and they are clearly non-exclusive and non-competitive because they are delivered through platforms such as networks, TV, and community bulletin boards. An increase in the number of people receiving health literacy services does not preclude others from receiving them, nor does the addition of a new audience for science popularization cause an increase in marginal costs. Concurrently, when one person receives popular health science services, it does not preclude or exclude others from obtaining the same quantity and quality of services, implying that health literacy service has the property of public goods.

Between public and private goods, there exists “quasi-public goods”, which are items that include the characteristics of both public and private goods, such as medical services, non-compulsory education, and some infrastructure that requires payment for use [31]. By considering health literacy services as an example, on the one hand, there is a certain degree of competition in the provision of health science services on-site (e.g., medical consultations, lectures, etc.), and when the number of services exceeds the limit of resource design, the number of venues, service facilities, and human resources required to provide them increases accordingly, and the marginal cost is not zero at this point. On the other hand, certain health science service channels are somewhat exclusive in terms of benefits, such as face-to-face health education in outpatient clinics and wards, where the time required for one patient to receive health education from health care staff may reduce the opportunities for other patients to receive health education. Therefore, health literacy services fall into the category of quasi-public goods.

As a quasi-public product, health literacy services cannot be provided entirely by the market, while the government, as the main provider, suffers from the dilemma of a single supply and limited resources [32]. To address the issue of whether the government or the market should exclusively provide public health services, Ostrom proposed polycentric governance theory, advocating a new governance model [33]. Polycentrism implies that public goods or services can and should have multiple producers, providers, and processors, producing a three-dimensional model of government, market, and society, in which citizens can make reasonable choices among various products and services according to their own needs. Their relationship is both mutual competition and cooperation. This avoids not only the limited capacity and single supply caused by the government monopoly but the “free-rider effect” caused by excessive privatization [34]. According to the questionnaire survey results, government departments, medical institutions, and media jointly provide health literacy services in response to the highest proportion of people’s choice, which widens the supply channels and modes of health science services, and paves a realization path to meet the growing demand for diversified health literacy services. During COVID-19 outbreak, health science services, led by government departments, voiced by authoritative experts, and delivered through online media, were widely recognized by the public. They assisted the public in the rational response to the sudden impact of epidemic, played a role in stabilizing people’s minds, and improved the scientific response capacity of the whole society.

The government plays a crucial role in public health literacy, from management to infrastructure construction, policy formulation, and implementation, and has unique advantages in terms of resources, channels, and talents [3]. Experts and scholars play the role of opinion leaders in health literacy services, [35] and media should cooperate with professional medical teams and adopt double-check mechanism in communication mode and content to effectively enhance their credibility and authority [36]. Concurrently, the government’s policy support and effective supervision must obtain more power space to play its maximum effectiveness.

### The overall supply of health literacy services is insufficient and unbalanced

With the continuous improvement of China’s economic level, people are increasingly concerned about health knowledge and pursue a healthy lifestyle, hoping to acquire a wealth of health information and knowledge to guide their daily work and lives. However, according to the questionnaire survey results, only 30% of township residents can often or always obtain necessary health literacy in a timely manner. The contradiction between the overall shortage of health service supply and the increasing demand remains prevalent. The coordination between health development and economic and social development must be strengthened [37].

The supply level of rural public goods is an important index to measure the development of rural economy and society [38]. Although the country has recently increased its investment in rural public goods, the main force has been on the economic construction of rural areas and productivity improvement. It is difficult to see immediate returns on some public goods, such as health education and agricultural research because they require significant investment and yield slow results. Some government officials, influenced by factors such as achievement views and economic interests, lack enthusiasm for their provision [39]. In addition, farmers’ demand for public goods is expressed through five levels of agency, from central government officials to provincial government officials to township government officials, which not only increases agency costs but also makes it easier for governments at all levels to perform their functions in a self-serving manner, without fully integrating the actual needs of rural people. Finally, this leads to agency failure, resulting in a disconnect or imbalance between supply and demand for rural public goods [40].

According to Health literacy Monitoring report of Chinese Residents (2018), urban residents had a health literacy level of 22.44%, while that of rural residents was 13.72% [41]. The Chinese government should prioritize health education services for rural residents, intensify health literacy, promote equalization of basic public services in the health field, maintain basic medical and health services of public welfare, and gradually reduce the differences between urban and rural, regional, as well as basic health services and health level between urban and rural residents to achieve universal health coverage and promote social equity [42].

In recent years, under the context of “equalization of basic public health services”, the supply capacity of health education directly or indirectly affects the effective performance of public health service functions and the improvement of residents’ overall health literacy, and supply capacity construction is critical for overcoming the rural health literacy service’s predicament. First of all, as direct service objects and beneficiaries of rural health literacy, rural citizens should have the right to decide their health services [43]. Governments at all levels should clarify their own responsibilities in supplying health education, investigating and analyzing the actual health needs of rural people, and creating a bottom-up mechanism for supplying public goods. In addition, the government should strengthen the development of special funds for health literacy service, the marketing operation, the expansion of financing channels, the promotion of multidisciplinary and multisectoral collaborative innovation, the exploration of standardized health literacy personnel training mode and channel construction mechanisms, the maintenance of a balance between urban and rural public products, and the tilt the development of health science education in rural areas in terms of policy support and financial support.

### The interpersonal communication channels of health literacy are prominent

According to survey’s results, WeChat public account (Circle of friends, WeChat public account), as a media platform with a high penetration rate, was selected by the masses as the most important way of obtaining health literacy through the network (84.27%), similar to the way of communication with acquaintances (46.9%). Both modes of communication reflect the value of “strong interpersonal communication”.

Lazarsfield’s “two-stage communication theory” (Shown in Table 9) holds that mass communication (primary communication) is more effective in communicating broadly, but interpersonal communication (secondary communication) is more effective at communicating deeply [44]. While mass communication plays a significant role in people’s access to information, it is primarily interpersonal communication that leads to changes in attitudes, values, and ultimately behavior [45]. Social media is an important source of information in people’s daily lives. In the United States, nearly 81% of teens and 74% of adults use some form of social media [46]. This kind of interpersonal communication mode based on friend relationship is two-way, with timely feedback and high interaction frequency, which can better address people’s social spiritual, and psychological requirements.

**Table 9:**
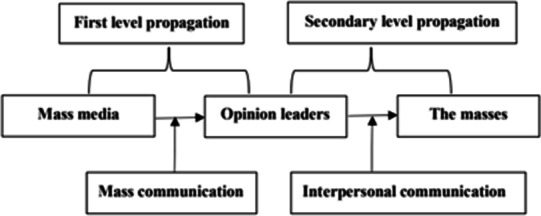
Lazarsfield’s two-stage propagation model

As mobile internet technology advances, interpersonal communication will unavoidably reconstruct a transmission power system. Using “retweeting”, “following”, “liking”, the public gains increasing control over content selection, to the point where it influences the selection of “first-level disseminators”. Such communication features are beneficial, as they encourage the media to raise their awareness of audience service and enhance pertinence and readability of health literacy works. However, in the absence of improved supervision and guidance, power promotion of “secondary transmission” will also have negative consequences.

Exaggeration and disinformation can flourish as a result of the mainstream media’s unbridled pandering to public tastes. According to the Crowd: A Study of the Popular Mind, “if an assertion is effectively repeated, there is no longer any objection to that repetition.” [47] . Interpersonal communication has inherent drawbacks in “checking” network information. Most citizens lack professional training, and their ability to detect rumors is insufficient, making them easily misled by rumors. If a rumor is amplified through interpersonal transmission, it is likely to become general consensus of society, increasing the difficulty of refuting the rumor. In mobile media environment, interactivity and convenience have increased dramatically, and the public does not require too much thinking when making judgments, spreading, sharing, and commenting, nor does it have to bear major responsibility in particular, which has made “emotional catharsis” a widespread phenomenon in the era of mobile media, along with accompanying incidents of “online public opinion violence” events, becoming more outstanding and prone to “moral judgment”.

### The popularity rate of network health literacy channel is high, and the trust rate is low

The results indicate that while online media is the most important channel for Wuhan citizens to obtain health literacy (85.29%), it is also the least trusted by the public.

According to the 47th Statistical Report of China’s Internet Development by CNNIC, as of December 2020, the number of China’s Internet users reached 989 million, an increase of 85.4 million compared with March 2020, and the Internet penetration rate reached 70.4% [48]. The structured measurement results of netizens’ demands for health literacy in China Science Popularization Internet Data Report 2020 reveal that health and medical care are the largest part of netizens’ popular science demand, with approximately 70% overall demand intensity and 60% overall demand width [17].

Contemporary health literacy is undergoing revolutionary changes. On the one hand, with the widespread use of mobile Internet, online health information and knowledge is accepted among more audiences due to the characteristics of more convenient information transmission and more diversified communication modes. On the other hand, in the social media era, everyone can become the subject of online communication, and opinion leaders are “ordinary people”. This results in mixed content and uneven quality of health literacy services [49]. To garner attention, some self-publishers place a greater emphasis on presentation and entertainment effect of the content than on the scientific nature of health literacy works, resulting in much health literacy works through the Internet having gorgeous and interesting appearance but lacking scientific thought, scientific spirit, and scientific connotation in their essence. Due to the lack of professional knowledge and logical thinking, netizens are easily attracted by “headline party” and are prone to retweet and share false information. In the absence of effective supervision and punishment mechanism, network media has become the least trusted source of health science information.

Health literacy service is a long-term activity to spread scientific and technical knowledge, scientific methods, scientific ideas, and scientific spirit in the field of health to the public through health literacy, aiming at cultivating public health literacy and assisting the public in self-management of their health [50]. Its objective is not only to impart basic knowledge and skills for living a healthy life to citizens but also to assist them in developing scientific thinking and conducting health management scientifically and effectively. Critical health literacy is more important than ever in an era of information overload and advanced development, particularly regarding the outbreak of infectious diseases and people’s growing expectations for health [51]. Through deductive reasoning, inductive argumentation, and other methods, critical thinking can help people see through phenomena to their essence, forming their own logical thinking and opinions on situations and events that are not easily influenced by emotion, public opinion, or others. Knowledge is constantly updated; even authoritative experts do not claim to possess the absolute truth. People should pay attention to evidence, learn to judge the credibility of different evidence, and set aside the inherent biases and existing positions to examine different opinions.

### Limitations

The key disadvantage of this study is that the sample size is insufficient, and the research was conducted mostly in Hubei Province, China, limiting its universality.

Furthermore, despite our best attempts to include as much material as feasible, publications with full texts in languages other than English or Chinese would have also limited the scope of this research. Future research should be undertaken to investigate larger samples globally and thoroughly, greater detail about the read and write models of health literacy could be studied.

## Conclusions

Globalization increases the risk of spreading known and emerging infectious diseases. Therefore, from a preventive medicine perspective, we must explore health content requirements of different groups and perform well in primary prevention (etiology prevention). To improve citizens’ self-awareness and self-control of their health, we must promote health literacy development, encourage each citizen to be responsible for his or her own health, guide people to establish a correct health perspective, form a healthy lifestyle, ecological environment, and social environment, and prevent diseases or public problems caused by diseases before they happen.

Based on the theory of public goods, through literature reading and questionnaire survey, this paper further emphasizes to develop a government-led multi-body joint supply of health literacy services. Combined with the survey results of public health literacy demand, for Hubei residents, the three most popular health literacy topics were nutrition, first aid knowledge, and mental health. Compare the health literacy service accessibility with different population, township area was significantly lower than urban areas. After summarizing the advantages and disadvantages of health literacy service under the background of network media era, we have tried to explore how to help the public identify the authenticity of network medical knowledge, establish scientific and effective health literacy services with high public participation.

## Supplementary Information

**Additional file 1.**

## Data Availability

The data-sets analyzed during the current study are available from the corresponding author on reasonable request.
