# Correction: How to improve public health literacy based on polycentric public goods theory: preferences of the Chinese general population

**DOI:** 10.1186/s12889-022-13417-0

**Published:** 2022-06-20

**Authors:** Yaxin Gao, Li Zhu, Zi Jun Mao

**Affiliations:** 1 Department of Gastrointestinal Surgery, Tongji Hospital, Tongji Medical College, Huazhong University of Science and Technology, Jie Fang Ave, Wuhan, No. 1095 China; 2 College of Public Administration, Huazhong University of Science and Technology, No 1037 Luau Road, Hongshan District, Wuhan, 430074 China; 3 Non-traditional Security Institute, Huazhong University of Science and Technology, Wuhan, 430074 China

**Correction: BMC Public Health 22, 921 (2022)**

**https://doi.org/10.1186/s12889-022-13272-z**

In the original publication of this [1] article table 8 and 9 were identical. The article has been updated with the correct version of Table 8. The correct Table 8 is also available in this correction article.

**Table 8:** Correlation analysis of health literacy contents

	1	2	3	4	5	6	7	8	9	10	11	12	13	14	15	16	17	18	19	20
1.Gender	1																			
2.Age	-.049^**^	1																		
3.Profession	.158^**^	.084^**^	1																	
4.Education	-.017	-.341^**^	-.238^**^	1																
5.Personal Income	-.182^**^	-.022	-.265^**^	.336^**^	1															
6.Current residence	-.010	-.033	.053^*^	-.233^**^	-.184^**^	1														
7.First aid knowledge	.045^*^	-.042^*^	.011	.084^**^	.026	-.058^**^	1													
8.Cosmetic surgery	.153^**^	-.183^**^	-.109^**^	.136^**^	.039	-.019	.284^**^	1												
9.Nutrition	.119^**^	-.005	.048^*^	.021	.002	-.051^*^	.627^**^	.256^**^	1											
10.Tumor	.095^*^	-.088^**^	-.013	.095^**^	.044^**^	-.044^*^	.593^**^	.336^**^	.576^**^	1										
11.Metabolic disease	.051^**^	.001	.005	.059^**^	.029	-.057^**^	.580^**^	.312^**^	.564^**^	.712^**^	1									
12.Cardiovascular disease	.052^**^	.064^**^	.015	.047^*^	.027	-.071^**^	.589^**^	.287^**^	.599^**^	.705^**^	.868^**^	1								
13.Mental health	.122^**^	-.042	.005	.047^*^	-.010	-.055^*^	.591^**^	.322^**^	.620^**^	.630^**^	.708^**^	.702^**^	1							
14.Children’ health	.105^**^	-.073^**^	.029	-.018	-.008	.029	.465^**^	.279^**^	.469^**^	.488^**^	.528^**^	.528^**^	.581^**^	1						
15.Facial features	.103^**^	-.046^*^	-.007	.055^*^	-.003	-.032	.507^**^	.384^**^	.556^**^	.566^**^	.608^**^	.600^**^	.669^**^	.498^**^	1					
16.Reproductive health	.045^*^	-.133^**^	-.013	.074^*^	.018	-.032	.483^**^	.397^**^	.482^**^	.616^**^	.614^**^	.604^**^	.618^**^	.539^**^	.688^**^	1				
17.Medical technology	.007	-.095^**^	-.064^**^	.079^*^	.035	-.009	.481^**^	.411^**^	.462^**^	.603^**^	.610^**^	.615^**^	.604^**^	.493^**^	.643^**^	.705^**^	1			
18.TCM	.083^**^	.004	-.015	.030	.006	-.015	.493^**^	.369^**^	.509^**^	.553^**^	.608^**^	.603^**^	.607^**^	.471^**^	.629^**^	.628^**^	.686^**^	1		
19.Infectious disease	.057^**^	-.011	.018	.034	.015	-.046^*^	.549^**^	.309^**^	.533^**^	.646^**^	.662^**^	.678^**^	.668^**^	.523^**^	.640^**^	.650^**^	.667^**^	.665^**^	1	
20.Venereal disease	.036	-.106^**^	-.017	.037	-.018	.007	.482^**^	.367^**^	.443^**^	.576^**^	.566^**^	.586^**^	.609^**^	.519^**^	.611^**^	.676^**^	.693^**^	.641^**^	.731^**^	1

*TCM *Traditional Chinese Medicine

^**^P<0.01; ^*^P<0.05
