# Supplementary material for: How to improve public health literacy based on polycentric public goods theory: preferences of the Chinese general population
Source: BMC Public Health. 2022 May 9;22:921. doi: 10.1186/s12889-022-13272-z (PMC9083483; doi:10.1186/s12889-022-13272-z)
Supplement: Supplementary file 1 — Additional file 1. [file 12889_2022_13272_MOESM1_ESM.docx]

**Appendix:**

**Attachment 1: Public health literacy service demand questionnaire**

**Public health literacy service demand questionnaire**

In order to cooperate with the national "14th Five-Year Plan" development plan, promote health science knowledge, improve residents' health literacy, understand the public's demand for health knowledge, and contribute to the continuous optimization of wuhan health science popularization service and the comprehensive construction of "Healthy China". Welcome to express your opinions and views.

**Basic information of the respondents**

1. Your gender: [single choice] *

| ○Man | ○Woman |  |  |  |  |  |  |
| --- | --- | --- | --- | --- | --- | --- | --- |

2. Your age： [single choice] *

| ○Under 18 | ○18~29 | ○30~39 | ○40~49 | ○50~59 | ○Above 60 |  |
| --- | --- | --- | --- | --- | --- | --- |

3. Your current occupation： [single choice] *

| ○Public servant |
| --- |
| ○Teacher |
| ○Technical professional |
| ○Ordinary worker |
| ○We Media Practitioner |
| ○Service Personnel |
| ○Freelancer |
| ○Farmer |
| ○Student |
| ○Stay-at-home mom/dad |
| ○Retiree |
| ○other |
| ○unemployed or underemployed |

4.Education [single choice] *

| ○Postgraduate and above |
| --- |
| ○Undergraduate |
| ○college |
| ○Senior high school |
| ○Junior high and below |

5. Personal monthly income [single choice] *

| ○below 2000RMB |
| --- |
| ○2000-5000 RMB |
| ○5000-10000 RMB |
| ○above 10000 RMB |

6. current residence [single choice] *

| ○City |
| --- |
| ○Villages and towns |

**Content requirements of health literacy service**

7. Your needs for different health topics [Matrix question] *

|  | No need | Small amount needed | Generally need | More needed | In great need |
| --- | --- | --- | --- | --- | --- |
| First aid knowledg | ○ | ○ | ○ | ○ | ○ |
| Cosmetic surgery | ○ | ○ | ○ | ○ | ○ |
| Nutrition | ○ | ○ | ○ | ○ | ○ |
| Tumor | ○ | ○ | ○ | ○ | ○ |
| Metabolic disease | ○ | ○ | ○ | ○ | ○ |
| Cardiovascular disease | ○ | ○ | ○ | ○ | ○ |
| Mental health | ○ | ○ | ○ | ○ | ○ |
| Children's health | ○ | ○ | ○ | ○ | ○ |
| Facial features | ○ | ○ | ○ | ○ | ○ |
| Reproductive health | ○ | ○ | ○ | ○ | ○ |
| Medical technology | ○ | ○ | ○ | ○ | ○ |
| TCM | ○ | ○ | ○ | ○ | ○ |
| Infectious disease | ○ | ○ | ○ | ○ | ○ |
| Venereal disease | ○ | ○ | ○ | ○ | ○ |

**Demand for health literacy service channels**

8. Whether you can find the health information and knowledge you need in time？ [single choice] *

| ○A. Always | ○B. Usually | ○C. Generally | ○D. Occasionally | ○E. Never |
| --- | --- | --- | --- | --- |

9. The main channel for you to obtain health information and knowledge is [Multiple choice] *

| □A. Network media |
| --- |
| □B. Communicate with acquaintances |
| □C. Community Publicity Board |
| □D. Hospital bulletin board |
| □E. Free clinic, lectures |
| □F. Television, radio |
| □G. Newspapers, magazines, books |
| □H. Outpatient and ward medical education |

The channel that you get health information and knowledge through network media is [Multiple choice] *( This question depends on the answer to question 9 option A)

| □A. WeChat |
| --- |
| □B. Weibo |
| □C. The popular science website |
| □D. Video websites or apps |
| □E. Search engine |
| □F. News website |
| □G. Short video apps |
| □H. Online Education Learning |
| □I. Network BBS |
| □J. Others _________________ |

10. Your trust degree in the following channels of access to health literacy services？[Matrix question] *

|  | Very distrustful | Distrustful | General trust | More trust | Very trust |
| --- | --- | --- | --- | --- | --- |
| Network media | ○ | ○ | ○ | ○ | ○ |
| Communicate with acquaintances | ○ | ○ | ○ | ○ | ○ |
| Community Publicity Board | ○ | ○ | ○ | ○ | ○ |
| Hospital bulletin board | ○ | ○ | ○ | ○ | ○ |
| Free clinic, lectures | ○ | ○ | ○ | ○ | ○ |
| Television, radio | ○ | ○ | ○ | ○ | ○ |
| Newspapers, magazines, books | ○ | ○ | ○ | ○ | ○ |
| Outpatient and ward medical education | ○ | ○ | ○ | ○ | ○ |

11. If you obtain health information and knowledge based on the Internet, the form of work you prefer is [Multiple choice]*

| □A. Articles with images |
| --- |
| □B. Short videos |
| □C. Audio |
| □D. Anime |
| □E. Live Streaming |
| □F. Video games |
| □G. Photography and painting |
| □H. Infographics (data visualization) |
| □I. Augmented reality |
| □J. Others _________________ |

**Demand for health** **literacy service**

12. Where do you prefer to get health literacy service： [Multiple choice] *

| □A. Ones' own home |
| --- |
| □B. Medical Institutions |
| □B1.Large general hospital |
| □B2.Community health service stations |
| □B3.Any hospital close to home |
| □C. Science Museum |
| □D. Community Public Places |
| □E. Campus Activity Center |
| □F. Enterprises |
| □G. Others _________________ |

13. Do you think the provider of health literacy service should be？ [single choice] *

| ○A. Community, sub-district, etc government departments | ○B. Medical institution | ○C. The media | ○D. Government departments, medical institutions, the media jointly provided |
| --- | --- | --- | --- |
